# 214. The impact of preservation fluid culture on graft site arteritis: a systematic review and metanalysis.

**DOI:** 10.1093/ofid/ofac492.291

**Published:** 2022-12-15

**Authors:** Matteo Rinaldi, Cecilia Bonazzetti, Milo Gatti, Natascia Caroccia, Giorgia Comai, Matteo Ravaioli, Maria Cristina Morelli, Pierluigi Viale, Maddalena Giannella

**Affiliations:** Infectious disease unit, IRCCS Policlinic S. Orsola, Bologna, Emilia-Romagna, Italy; Department of Medical and Surgical Sciences, Alma Mater Studiorum University of Bologna, Bologna, Emilia-Romagna, Italy; Pharmacology unit, IRCCS Policlinic S. Orsola, Bologna, Emilia-Romagna, Italy; Department of Medical and Surgical Sciences, Alma Mater Studiorum University of Bologna, Bologna, Emilia-Romagna, Italy; Nephrology, Dialysis and Renal Transplant Unit, IRCCS Policlinic S. Orsola, Bologna, Emilia-Romagna, Italy; Department Medical and Surgical Sciences, General Surgery and Transplant Unit, IRCCS Policlinic S. Orsola, Bologna, Emilia-Romagna, Italy; Internal Medicine Unit for the Treatment of Severe Organ Failure, IRCCS Policlinic S. Orsola, Bologna, Emilia-Romagna, Italy; Infectious Diseases Unit, Department of Medical and Surgical Sciences, Policlinico Sant'Orsola Malpighi, University of Bologna, Bologna, Italy, Bologna, Emilia-Romagna, Italy; Department of Medical and Surgical Sciences, Alma Mater Studiorum University of Bologna, Bologna, Emilia-Romagna, Italy

## Abstract

**Background:**

The role of culturing the graft preservation fluid (PF) is controversial and its impact on graft arteritis development remains unclear.

**Methods:**

Systematic literature search retrieving all-languages observational studies comparing SOT recipients with culture positive PF versus culture negative PF. Graft site arteritis within 180 days from transplant was selected as primary outcome. Data were independently extracted using a pre-specified form, and quality of included studies was independently assessed according to ROBINS-I tool for observational studies. Meta-analysis was performed using Mantel-Haenszel random-effect models.

**Results:**

2,492 articles were screened, and twenty-one observational studies (N=2,208 positive PF vs. 4,458 negative) were included in the meta-analyses. Among positive PF, 857 (38.8%) were classified as high-risk group pathogens (fungi, Gram negative, *Enterococcus spp*., beta-hemolytic streptococci and *Staphylococcus aureus*) and 1,351 (61.2%) as low-risk (mostly coagulase negative Staphylococci) pathogens. A significant higher risk of graft arteritis was found in SOT recipients with a PF yielding a high-risk pathogen (odds ratio [OR] 18.43, 95%CI 7.83-43.40), with low heterogeneity (I²=2.24%). Similar results were found considering separately high-risk bacteria (OR 12.02, 95%CI 4.88-29.60) and fungi (OR 71.00, 95%CI 28.07–179.56), with no heterogeneity (I²=0%), and in the subgroup analyses of liver (OR 16.78, 95%CI 2.95–95.47) and kidney (OR 19.90, 95%CI 4.78–82.79) recipients. However, data about diagnostic features of graft arteritis were very limited, indeed for only 11 of the 93 events histological or microbiological results were reported.

**Conclusion:**

Our results may support the performance of PF culturing and a preemptive diagnostic or therapeutic management upon isolation of high-risk pathogen. Further studies based on reliable diagnosis of graft arteritis are needed to confirm our findings.

**Disclosures:**

**All Authors**: No reported disclosures.

